# Augmenting subnetwork inference with information extracted from the scientific literature

**DOI:** 10.1371/journal.pcbi.1006758

**Published:** 2019-06-27

**Authors:** Sid Kiblawi, Deborah Chasman, Amanda Henning, Eunju Park, Hoifung Poon, Michael Gould, Paul Ahlquist, Mark Craven

**Affiliations:** 1 Department of Computer Sciences, University of Wisconsin, Madison, WI, USA; 2 Department of Biostatistics and Medical Informatics, University of Wisconsin, Madison, WI, USA; 3 Wisconsin Institute for Discovery, University of Wisconsin, Madison, WI, USA; 4 Department of Oncology, University of Wisconsin, Madison, WI, USA; 5 Institute for Molecular Virology, University of Wisconsin, Madison, WI, USA; 6 Morgridge Institute for Research, Madison, WI, USA; 7 Microsoft Research, Redmond, WA, USA; 8 Howard Hughes Medical Institute, University of Wisconsin, Madison, WI, USA; University of Chicago, UNITED STATES

## Abstract

Many biological studies involve either (i) manipulating some aspect of a cell or its environment and then simultaneously measuring the effect on thousands of genes, or (ii) systematically manipulating each gene and then measuring the effect on some response of interest. A common challenge that arises in these studies is to explain how genes identified as relevant in the given experiment are organized into a subnetwork that accounts for the response of interest. The task of inferring a subnetwork is typically dependent on the information available in publicly available, structured databases, which suffer from incompleteness. However, a wealth of potentially relevant information resides in the scientific literature, such as information about genes associated with certain concepts of interest, as well as interactions that occur among various biological entities. We contend that by exploiting this information, we can improve the explanatory power and accuracy of subnetwork inference in multiple applications. Here we propose and investigate several ways in which information extracted from the scientific literature can be used to augment subnetwork inference. We show that we can use literature-extracted information to (i) augment the set of entities identified as being relevant in a subnetwork inference task, (ii) augment the set of interactions used in the process, and (iii) support targeted browsing of a large inferred subnetwork by identifying entities and interactions that are closely related to concepts of interest. We use this approach to uncover the pathways involved in interactions between a virus and a host cell, and the pathways that are regulated by a transcription factor associated with breast cancer. Our experimental results demonstrate that these approaches can provide more accurate and more interpretable subnetworks. Integer program code, background network data, and pathfinding code are available at https://github.com/Craven-Biostat-Lab/subnetwork_inference

## Author summary

There is a multitude of publicly available databases that contain information about biological entities (i.e., genes, proteins, and other small molecules) as well as information about how these entities interact together. However, these databases are often incomplete. There is a wealth of information present in the text of the scientific literature that is not yet available in these databases. Using tools that mine the scientific literature we are able to extract some of this potentially relevant information. In this work we show how we can use publicly available databases in conjunction with the information extracted from the scientific literature to infer the networks that are involved in specific biological processes, such as viral replication and cancer tumor growth.

## Introduction

An important and pervasive type of analysis in systems biology research is to characterize the set of molecular entities and interactions that are involved in a biological process or response of interest. This type of analysis, which we refer to as *subnetwork inference*, takes as input background knowledge describing potentially relevant entities and interactions (a network), along with experimental data characterizing the relevance of entities to the response of interest. It returns as output a subset of the entities and interactions (a subnetwork) that are predicted to be centrally involved in the response. This approach has been shown to lend insight and make accurate predictions in a wide range of biological applications. However, it suffers from several key limitations that arise due to the immense search space, and the reliance of the approach on curated databases of interactions. In this work, we explore several ways in which the subnetwork inference approach can be augmented with information that is automatically elicited from the scientific literature, and we empirically demonstrate that such literature extracted information can lead to more accurate and interpretable subnetworks.

**Fig 1:**
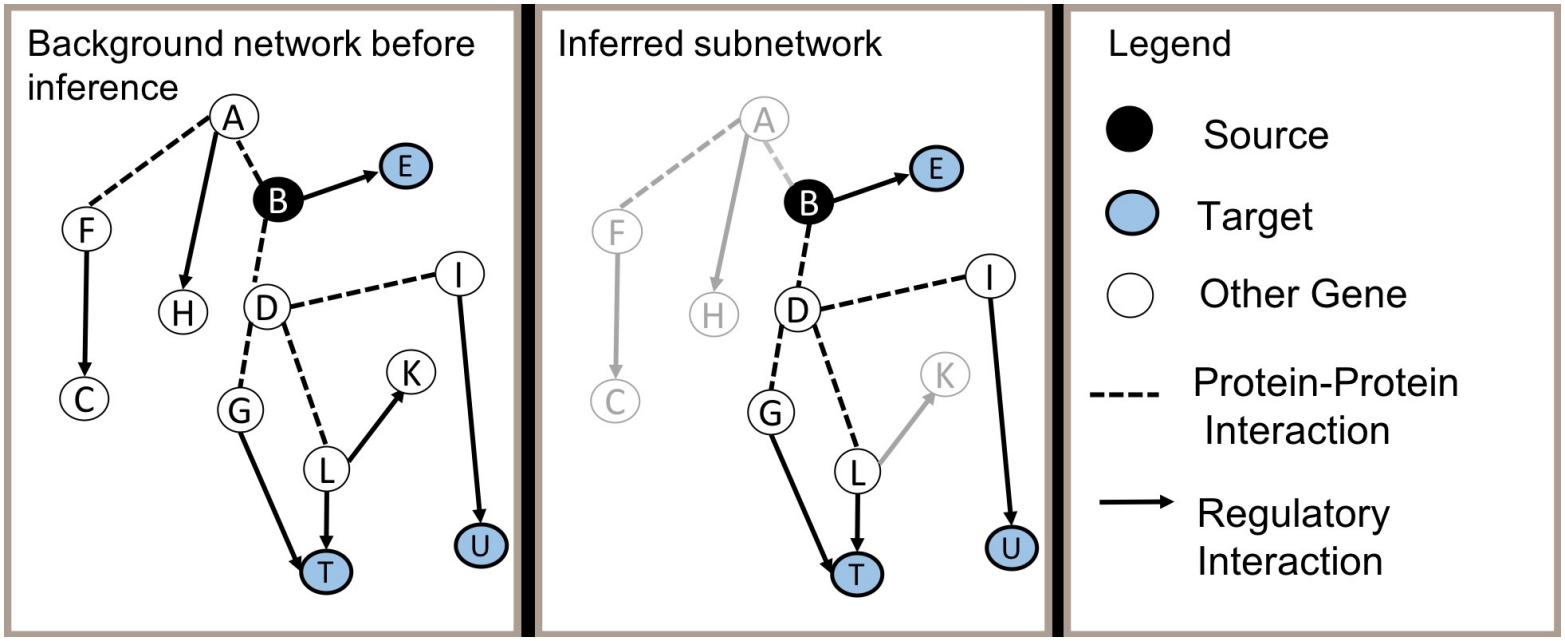
Overview of subnetwork inference task. The first panel shows a background network composed of molecular entities and known interactions among them. A subset of the entities are designated as source nodes (black), and a subset as target nodes (blue). White nodes show elements that are not considered source or target nodes. The second panel displays an inferred subnetwork which includes only a subset of the entities and interactions in the background network. Elements of the background network that are not included in the inferred subnetwork are greyed out.

Fig 1 provides an overview of the subnetwork inference task. One of the inputs to a subnetwork-inference approach is a *background network* consisting of (i) entities such as genes/proteins and complexes, and (ii) intracellular interactions such as protein-protein interactions, protein-DNA interactions, protein constituents of a complex, etc. The background network is commonly assembled by integrating interactions from publicly accessible, curated databases such as BioGRID [1] and Reactome [2]. Note that although each of these interactions is believed to occur in some cellular context, many of them may not be involved in the response of interest. When representing the background network as a graph, the nodes correspond to entities and the edges correspond to interactions. The other inputs for the task typically are sets of *source* and *target* nodes. These sets might be identified by experimental data or they might be defined using background knowledge. The computational task of subnetwork inference is to (i) identify a subset of edges and nodes in the graph that enable the sources to be connected to the targets, while (ii) adhering to constraints that specify required properties of the subnetwork (e.g., there must be at least one path from each source to a target), and (iii) optimizing an objective function that describes desirable properties of the subnetwork (e.g., it must be minimal in some sense).

Here, we consider two applications of subnetwork inference that are representative of the general task. The first application is to infer the host-cell subnetwork that is exploited by HIV during virus replication. In this analysis, the source nodes are host genes that have been identified as playing a significant role in viral replication via RNAi assays that systematically knock the genes down. The target nodes are HIV proteins. The goal of the inference task is to determine a subnetwork that explains how the RNAi-identified genes might be affecting HIV replication [3, 4].

The second application we consider is focused on characterizing a nuclear receptor, NR2F1, which is an important factor mediating the activity of the Mcs1a Mammary Carcinoma Susceptibility locus, and may have therapeutic relevance to triple-negative breast cancer [5]. In this analysis, NR2F1 is the sole source node, and the target nodes correspond to the genes that are differentially expressed when NR2F1 is overexpressed. The goal of the subnetwork inference task in this case is to identify the regulatory interactions that link NR2F1 to its downstream-regulated genes.

Although the subnetwork inference approach has proven to have significant value in a broad range of applications [6, 7, 8, 9, 10, 11, 12, 13, 14, 15, 16, 17], it suffers from a number of key limitations. One of these is that the ability of the approach to identify a subnetwork that provides an accurate characterization of the response of interest is limited by the completeness and accuracy of the interactions represented in the background network. This, in turn, is determined by the completeness and accuracy of the curated data sources from which the background network is assembled. Although curated databases of molecular interactions tend to have high accuracy, it is well known that they capture only a fraction of the interactions that actually occur in cells. Moreover, these data sources are limited by what they have deemed eligible for inclusion, which are typically experimentally verified direct physical interactions. Few resources include indirect interactions, for example. Another limitation of the subnetwork-inference approach is that the putatively relevant entities—the sources and targets—may also be incomplete. Consider, for example, the task of identifying host genes involved in HIV replication from RNAi experiments. We know that RNAi screens in this context are likely to result in many false negatives, and even with multiple screens, we are likely to not detect many of the involved host genes [18]. A third limitation of the approach is that inferred subnetworks may be complex and hard to understand. This may be the case simply because the number of relevant nodes is large. For example, in the HIV application, more than a thousand genes are detected by the experimental screens as being relevant to HIV replication.

**Fig 2:**
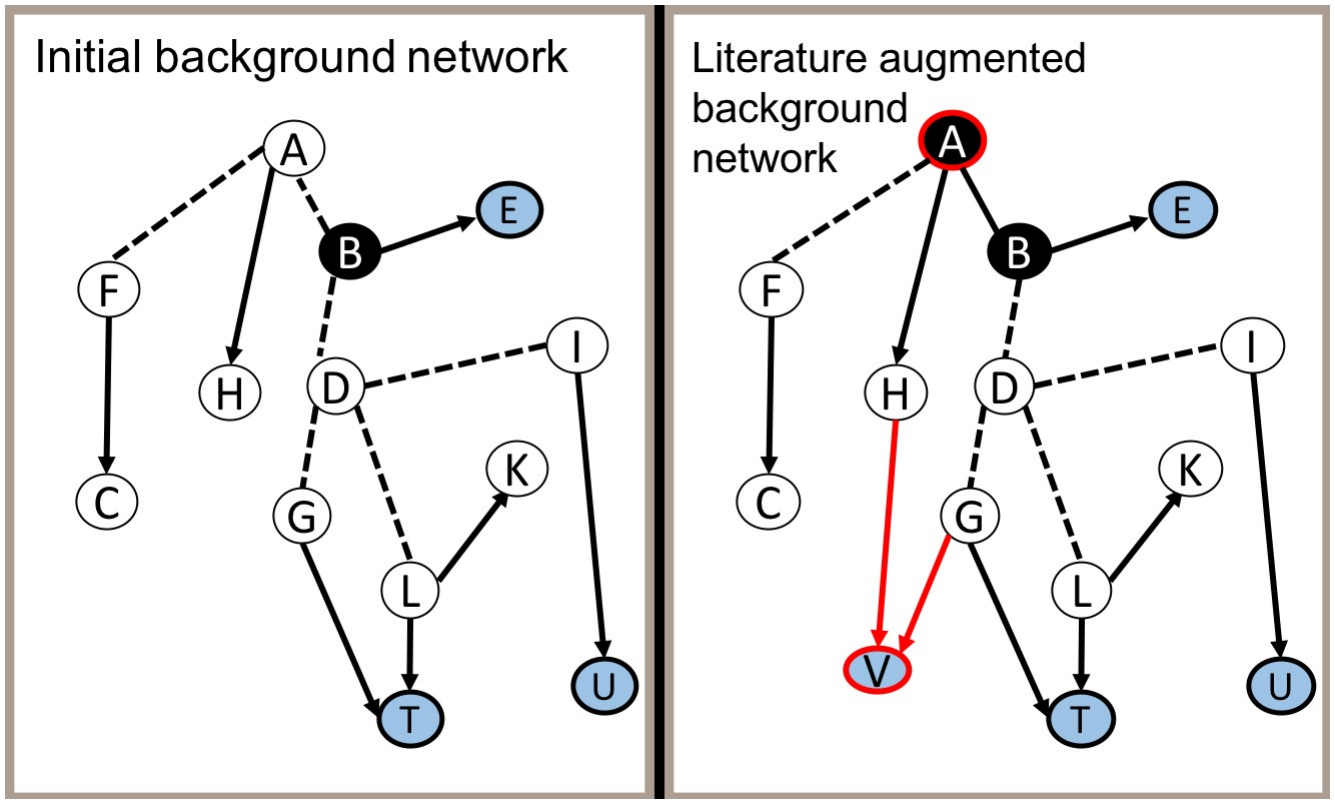
General representation of a background network augmented with information from scientific literature. The background network is composed of source nodes (black), target nodes (blue), and interactions (dashed and solid lines). Elements outlined in red represent information extracted from the scientific literature. In this case, the literature has been used to identify another source node (A), an additional target (V), and two additional interactions.

**Fig 3:**
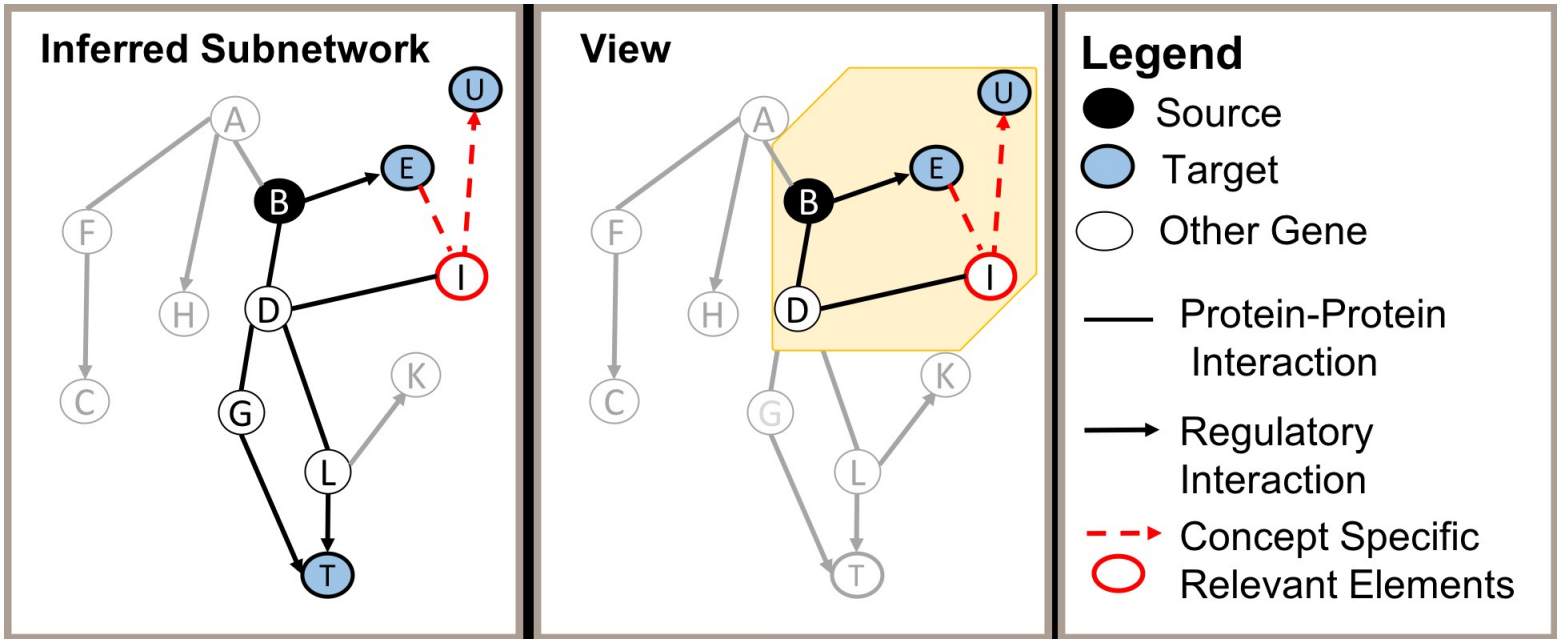
General representation of a view generated from a subnetwork. The first panel shows an inferred subnetwork. The second panel shows a view (yellow background) which delineates a set of nodes and interactions that are related to a concept of interest. The inferred subnetwork and view are composed of source nodes (black), target nodes (blue), and interactions (dashed and solid lines). Elements outlined in red represent entitities and interactions used as the basis of the view (i.e., those most closely linked to the concept).

We argue that subnetwork inference methods are hindered by failing to take advantage of the wealth of knowledge that is represented only in the scientific literature, as opposed to structured databases. The hypothesis driving the research presented here is that we can more accurately characterize responses of interest by automatically extracting and leveraging information from the scientific literature. In this article, we investigate the use of literature-extracted information to (i) augment the set of nodes identified as sources in a subnetwork inference task, (ii) augment the set of interactions used in the background network, and (iii) support targeted browsing of a large inferred subnetwork by computing *views* of the subnetwork which consist of nodes and edges that are closely related to concepts of interest. Whereas an inferred subnetwork represents a process of interest (e.g., HIV replication), views can be used to identify and inspect more specific concepts within the inferred subnetwork (e.g., membrane scission). Fig 2 illustrates the first two of these augmentations, while Fig 3 represents the task of generating a view of a subnetwork.

## Methods

In this section, we describe two subnetwork-inference applications considered in this article that we address using integer programming (IP). We also describe three ways in which we have augmented this approach by using literature-extracted information.

### HIV host-virus interaction task

One approach to characterizing the host cellular machinery that is hijacked by a virus is to systematically suppress host gene products using techniques such as RNA interference [19], mutant libraries [20], or CRISPR/Cas9 [21]. Typically, these genome-wide screens identify a large number of host genes, which we refer to as *hits*, whose loss has a significant effect on the virus. However, the screens themselves do not reveal how the gene products of these hits are organized into the pathways that modulate the virus. Moreover, they may fail to detect a large number of host genes that are involved in the process [18]. Here, we consider the computational task of inferring directed subnetworks that hypothesize the pathways through which each hit modulates viral replication and also posit additional host genes that are involved in viral replication. Our methodology is based on previous work we have done using an integer program (IP) to infer the relevant subnetwork [3, 4].

**Fig 4:**
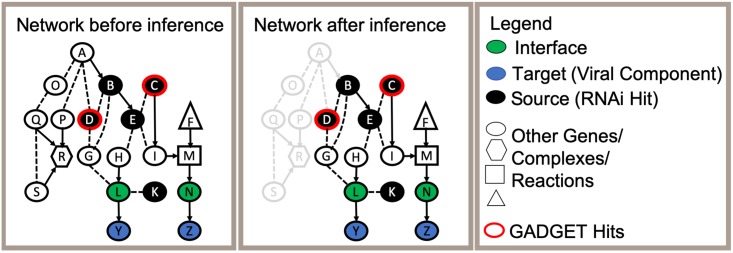
Overview of the HIV host-virus subnetwork inference task. The first panel displays a background network composed of source RNAi hits (black), interface nodes (green), and interactions (dashed and solid lines). Each interface is connected to a target viral component (blue). We can optionally augment the set of sources by using genes identified by GADGET as being relevant to HIV replication (red outline). The second panel displays an inferred subnetwork. Elements of the background network that are not included in the inferred subnetwork are shown in light grey.

In this task, which is illustrated in Fig 4, the source nodes are the genes that are found to be essential to HIV replication when knocked down using RNAi (i.e., the hits from these studies), and the target nodes are viral components which can be reached in the network via host factors that are known to interact directly with them. We refer to these host factors that directly interact with viral components as *interfaces*. The goal is to infer a subnetwork consisting of paths, each of which is a linear chain of interactions that begins with a hit (a source) and ends with a host gene product that directly interacts with a viral protein (a target).

The hits come from five RNAi screening studies that have identified human genes involved in HIV replication [22, 23, 24, 25, 26]. The union of the hit sets contains 1,178 host genes that act as source nodes in our subnetwork inference process. We compile a set of human-HIV interface proteins from the NCBI Human HIV-1 Protein Interaction Database [27] and BioGrid [1]. From these databases, we select as interfaces those host gene products that have a direct physical interaction with an HIV protein. In total, the background network contains 1,693 interfaces, 195 of which are also RNAi hits.

A background network is assembled from publicly accessible databases. We retrieve protein-kinase interactions, post-translational modification, and protein-protein interactions from a variety of sources [1, 28, 29, 30]. Protein complexes and reactions are taken from the Reactome collection of curated pathways [2]. The network is represented as a partially-directed graph. Each protein-protein interaction is represented as an edge in the graph, with directed interactions (such as kinase-substrate) represented as directed edges. Protein complexes are represented as separate nodes, with directed edges linking constituent genes to the complex. The reactions are separate nodes in the graph, with directed edges coming in from their inputs and catalysts, and directed edges going out to their products. The inputs and outputs to the reactions may be molecules, gene products, or protein complexes, and the catalysts may be gene products or protein complexes. The background network contains a total of 197,184 edges among 22,192 nodes, which include 9 HIV genes and 14,534 human genes. The remaining nodes are complexes, reactions, and small molecules.

The first step in our subnetwork inference approach is to generate a set of candidate paths. Using the hits as source nodes, we use a depth-first traversal to find all directed acyclic paths that lead from the source nodes to the targets. The search is conducted to a maximum depth of three interactions, so all paths have at most four nodes. All paths have the same directionality, going from the source node to an interface target node.

We refer to paths, nodes, and edges that are included in an inferred subnetwork as being *relevant*. The integer program identifies a subnetwork by determining values for a set of binary variables that represent the relevance of paths, edges, and nodes. The relevance of a path *p* is represented with a binary variable *σ*_*p*_, which takes the value of 1 if the particular path is included in the subnetwork, and 0 otherwise. The relevance of an edge *e* is represented by the binary variable *x*_*e*_, which takes a value of 1 if the edge is in at least one relevant path, and 0 otherwise. The relevance of a node *n* is represented by the binary variable *y*_*n*_, which takes a value of 1 if the node is present in any relevant paths, and 0 otherwise.

The integer program determines the settings for these variables using the objective functions and constraints shown in Table 1. We denote the set of nodes as N, the subset of source nodes (i.e., hits) as $N^{H}$, and the subset of interface genes as $N^{I}$. $N^{U}$ is the set of *unconfirmed* genes—those that are neither hits nor interfaces. $N^{C}$ is the set of protein complexes and $N^{R}$ represents reactions. E refers to the set of edges, E(n) refers to the edges that touch a particular node *n*, and N(e) represents the nodes that are involved in edge *e*. The set of paths is denoted as P. E(p) represents all edges in a given path *p*, N(p) represents all nodes that are contained within path *p*, P(n) represents the set of paths involving node *n*, and P(e) represents the set of paths involving edge *e*.

**Table 1:** Objective functions and constraints for the host-virus integer program. The left column describes each objective function and constraint. The right column provides the mathematical formulation for each.

Description	Mathematical Formulation
**Objective Functions:**	
(1) Maximize sum of relevant node scores	$max\sum_{n\in N^{U}}score\left(n\right)y_{n}$
(2) Maximize paths	$max\sum_{p\in P}\sigma_{p}$
**Constraints:**	
Limit the number of unconfirmed nodes determined to be relevant	$\sum_{n\in N^{U}\;}y_{n}\leq \delta$
Edges in relevant paths must be relevant	$\forall e\in E\;x_{e}\leq \sum_{p\in P(e)}\sigma_{p}$
	$\forall p\in P\;\forall e\in E\;\sigma_{p}\leq x_{e}$
Nodes in relevant edges must be relevant	$\forall n\in N\;y_{n}\leq \sum_{e\in E(n)}x_{e}$
	$\forall e\in E\;\forall n\in N\;x_{e}\leq y_{n}$
Nearly all hits must be relevant	$\sum_{n\in N^{H}\;}y_{n}\geq \left(1-\epsilon \right)\left|N^{H}\right|$
Nearly all interfaces must be relevant	$\sum_{n\in N^{I}\;}y_{n}\geq \left(1-\epsilon \right)\left|N^{I}\right|$
Majority of protein subunits must be relevant for complex to be relevant	$\forall c\in N^{C},\beta \leq 1\;\sum_{n\in N(c)\;}y_{n}+\left(1-y_{c}\right)|(N\left(c\right)|\geq \beta |N\left(c\right)|$
	$\beta =2\frac{|N^{H}\cup N^{I}|+\delta }{20000}$
	$\forall c\in N^{C},\forall e=\left(n,c\right)\in E\;x_{e}\leq y_{c}$
Reaction substrates and products must be relevant for reaction to be relevant	$\forall r\in N^{R},\forall n\in N\left(r\right)\;y_{r}\leq y_{n}$

The two objective functions are optimized sequentially. We use the first objective function to select the relevant nodes, and the second to identify all possible paths among those nodes. The rationale for the second objective function is to avoid arbitrarily selecting the paths that are included in the subnetwork. Instead, we opt to include all paths that satisfy the other constraints and connect the selected nodes. We know that the set of hits is incomplete given that RNAi screens typically have many false negatives [18], and thus we would like to predict which other host genes are involved. However, we need a way to limit the size of the inferred subnetwork so as to not include everything. In our IP, we control the size of the inferred subnetwork by constraining the number of unconfirmed genes that can be included in the subnetwork, and use the first objective function to include those that maximize predicted relevance scores which are computed using a diffusion kernel [31]. The intuition behind this method is that each hit carries some amount of weight that is partially diffused out via its neighbors in the background network. Each node in the network thereby receives a weight according to its proximity and connectivity to the set of hits. After selecting which nodes are to be included in the subnetwork, the second objective function then maximizes the inclusion of paths from sources to targets that use these nodes. We solve the IP using a branch-and-cut method [32].

Due to the fact that there are multiple solutions that satisfy all the constraints and maximize the objective functions, we generate an ensemble of solutions and then return a consensus subnetwork characterizing the nodes and edges that occur with high frequency in the ensemble. We first construct 100 subsampled data sets by holding aside 25% of the hits and interfaces in each, thus treating them as unconfirmed genes. To construct the ensemble, we run the IP independently on each subsampled data set.

### Augmenting HIV host-virus subnetwork inference with literature-extracted information

As mentioned above, the intersection among the hit sets identified by the RNAi knockdown screens is quite small. Prior research has indicated that this is due to the screens having many false negatives, and thus the number of genes involved in viral replication is likely to be much larger than even the number represented by the union of the HIV RNAi screens [18]. To address this limitation, we explore an approach that augments our hit set with additional genes that are associated with HIV replication in the scientific literature. More generally, we can think of this as an approach for augmenting a set of sources (or targets).

Our approach is based on a web-based tool called GADGET [33] that we have developed. GADGET identifies and ranks genes and metabolites that are associated in the biomedical literature with given queries. The queries may specify phenotypes, disease states, drugs, genes, processes, and other concepts that are expressible in a standard search-engine query language. GADGET ranks the genes/metabolites according to their association with the query. It is able to use several different ranking criteria, but the default criterion is $F_{1}=\frac{2\times \text{precision}\times \text{recall}}{\text{precision}+\text{recall}}$ where precision and recall are defined as follows. Let *A*_*g*_ represent the set of abstracts mentioning gene *g*, *A*^*q*^ be the set of abstracts matching query *q*, and $A_{g}^{q}$ be the set of abstracts that both mention gene *g* and match the query *q*. We define adjusted precision as $\frac{|A_{g}^{q}|}{|A_{g}|+10}$. The adjusted precision criterion includes a “pseudocount” of 10 in the denominator in order to bias the measure towards those genes for which there is more evidence indicating their association with the query. We define recall as $\frac{|A_{g}^{q}|}{|A^{q}|}$, i.e. the fraction of the abstracts matching the query that also mention gene *g*. *F*_1_ is the default ranking criterion in GADGET since it prefers genes whose associated literature is both specific to the query ands cover many of the query-relevant abstracts.

To augment our set of hits in the HIV subnetwork-inference task, we query GADGET for “HIV” which returns an additional 738 human genes that appear in two or more query-matching abstracts and which were not already in our set of interfaces or RNAi-screen identified hits. Instead of employing GADGET’s ranking functions in this analysis, we simply use all 738 of these genes. In our view-generation experiments, which are described shortly, we make use of GADGET’s ranking capability. We add these 738 genes to the IP as additional sources, and generate an ensemble of subnetworks as described above. This idea is illustrated in Fig 4 where some of the nodes (those with a red border) have been determined to be sources by GADGET.

### NR2F1 subnetwork inference task

**Fig 5:**
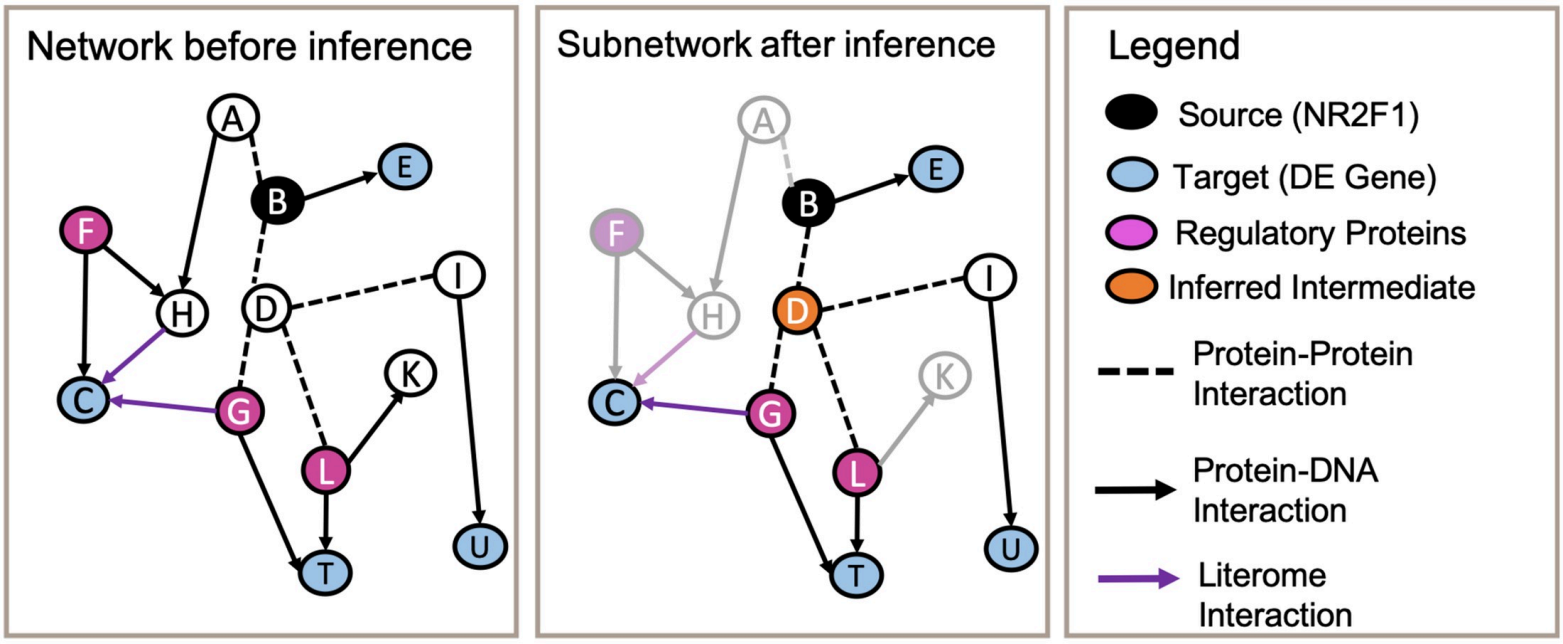
Overview of the NR2F1 subnetwork inference task. The first panel displays a background network composed of the source NR2F1 (black), target DE genes (blue), and interactions (dashed and solid lines). Transcription factors are identified as bright magenta nodes. The background network is augmented by a set of Literome interactions (purple arrows). The second panel displays the inferred subnetwork. Orange nodes show elements that are not considered source, transcription factors, or target nodes; we refer to these as intermediate nodes in the inferred subnetwork.

In the second application we consider, our objective is to connect a nuclear receptor known as NR2F1 (the source node) to a list of genes that are differentially expressed when NR2F1 is over-expressed (the target nodes). Prior studies suggest NR2F1’s potential as a therapeutic agent in triple negative breast cancers (TNBC) since several lines of evidence indicate that NR2F1 may act as a tumor suppressor, given its association with decreased proliferation and less aggressive clinical subtypes [5, 34]. The subnetwork-inference task we consider here is to find paths connecting NR2F1 to as many differentially expressed (DE) genes as possible, while identifying other genes that mediate the regulation of the DE genes. An overview of this task is shown in Fig 5.

RNA-Seq was used to identify a set of genes that were differentially expressed when NR2F1 was overexpressed in a TNBC cell line. This set of 340 DE genes served as the targets in our analysis. To assemble the background network, protein-protein interactions were gathered from HPRD [30] and BioGRID [1]. Protein-DNA interactions were gathered from MCF-7 ChIP-chip data collected by Kittler et al. [35]. Although MCF-7 cells do not represent TNBC, this was the closest cell line for which ChIP-chip data was available and our goal was to generously include candidate interactions that might potentially be relevant to TNBC. Additional protein-DNA interactions relevant to the differentially expressed genes were identified using the ENCODE ChIP-Seq Significance Tool (encodeqt.simple-encode.org) [36]. The input to the ENCODE tool consisted of our set of DE genes The tool searches a 5000bp upstream and downstream window in all available cell lines in order to find potential regulatory proteins associated with a given set of genes. The complete background network consisted of 349,149 interactions (171,789 protein-protein and 132,662 protein-DNA interactions) and 14,874 genes/proteins.

Using NR2F1 as the source node, a depth-first traversal was used to find all directed acyclic paths that lead from NR2F1 to the DE genes. Each path is required to end in a direct protein-DNA interaction and the search was conducted to a maximum depth of three interactions, so all paths had at most four nodes. All paths have the same directionality, going from the NR2F1 source node to a DE target node.

Given these paths, we infer an NR2F1 subnetwork by solving a series of integer programs. The role of the IP approach is to select a subset of the paths (and hence interactions and nodes) from the background network that reach as many of the DE genes as possible, while being parsimonious about incorporating non-DE genes, and taking into account RNA-Seq expression levels indicating the relevance of each included node. To incorporate RNA-Seq data into this process, normalized counts per million values were obtained from edgeR [37] and were used to weight nodes for selection, with the requirement that incorporated genes be expressed in MDA-MB-468 cells, which is a triple negative breast cancer cell line. The rationale for the final objective function is to avoid arbitrarily selecting the edges that are included in the subnetwork. Instead, we opt to include all edges that satisfy the other constraints and connect the selected nodes.

A description of the constraints and objective functions used in the IPs is shown in Table 2. The objective functions are optimized in sequence, with the solution to each subsequently being incorporated as a constraint before the next one is optimized. We denote the set of nodes as N where each node, *n*, represents either a protein or a target gene. $N^{S}$ is a single element set containing the source node, NR2F1, and $N^{T}$ is the set of DE genes. $N^{I}$ represents the remaining nodes that are not the source node or targets. The set of edges E represents both the undirected (protein-protein interactions) and directed (protein-DNA interactions) edges. E(n) refers to the edges that touch a particular node *n*, and $E^{\to }\left(n\right)$ refers to the set of edges directed into node *n*. N(e) represents the nodes that are involved in edge *e*. The set of paths is denoted as P. E(p) represents all edges in path *p*. N(p) represents all nodes that are contained within a specific path. P(n) represents the set of paths involving node *n*, and P(e) represents the set of paths involving edge *e*. The RNA-Seq determined score for a node *n* is given by *r*_*n*_.

**Table 2:** Objective functions and constraints for the NR2F1 integer program. The left column describes each objective function and constraint. The right column provides the mathematical formulation for each.

Description	Mathematical Formulation
**Objective Functions:**	
(1) Maximize number of DE genes reachable	$max\sum_{n\in N^{T}\;}y_{n}$
(2) Minimize number of intermediates	$min\sum_{n\in N^{I}\;}y_{n}$
(3) Maximize sum of included node scores	$max\sum_{n\in N^{I}\;}y_{n}r_{n}$
(4) Maximize edges included	$max\sum_{e\in E}x_{e}$
**Constraints:**	
Require at least one path to each reachable DE gene	$\forall n\in N^{T}\;y_{n}\leq \sum_{p\in P(n)}\sigma_{p}$
Edges in relevant path must be relevant	$\forall e\in E\;x_{e}\leq \sum_{p\in P(e)}\sigma_{p}$
	$\forall p\in P\;\forall e\in E\;\sigma_{p}\leq x_{e}$
Nodes in relevant edge must be relevant	$\forall n\in N\;y_{n}\leq \sum_{e\in E(n)}x_{e}$
	$\forall e\in E\;\forall n\in N\;x_{e}\leq y_{n}$
DE genes must be connected by directed edge	$\forall n\in N^{T}\;y_{n}\leq \sum_{e\in E^{\to }\left(n\right)}x_{e}$
Non-DE genes must have no incoming directed edge	$\forall n\in N^{I}\;0=\sum_{e\in E^{\to }\left(n\right)}x_{e}$

The integer program identifies a subnetwork by determining values for a set of binary variables that represent the relevance of paths, edges, and nodes. The relevance of a path *p* is represented with a binary variable *σ*_*p*_, which takes the value of 1 if the particular path was predicted to be included, and 0 otherwise. The predicted relevance of an edge is represented by the binary variable *x*_*e*_, which takes a value of 1 if the edge was in at least one relevant path, and 0 otherwise. The predicted relevance of a node is represented by the binary variable *y*_*n*_. The variable receives a value of 1 if it is present in any relevant paths, and 0 otherwise.

We use the IP approach to infer an ensemble of subnetworks from subsampled datasets and then construct a consensus subnetwork as we did in the host-virus analysis.

### Augmenting NR2F1 subnetwork inference with literature-extracted information

As previously discussed, one of the key limitations of the standard subnetwork-inference approach is that it relies on existing, structured databases of interactions which may be highly incomplete. Although the interactions present in publicly available databases allow us to generate paths connecting NR2F1 to many of the DE genes, we are not able to reach all of them. To address this limitation, we exploit information extracted automatically from the scientific literature. There is a large body of prior work addressing the tasks of extracting binary relations and more complex events from text sources [38, 39, 40], as well as efforts to assemble biological networks from these extracted relationships [41, 42]. However, these information-extraction methods have not been previously used to complement a background network in a subnetwork-inference application.

In order to augment our existing interactions, we use the Literome system [43, 44] which is able to extract regulatory interactions from both abstracts and the full-text of articles in the scientific literature. By querying Literome for our DE genes, we retrieved interactions extracted from the text and added them to our background network. In total, 44,879 additional Literome interactions were added. In our IP, we treat these regulatory interactions in the same way as our protein-DNA interactions and otherwise run the IP in the same way.

### Generating a view of an inferred subnetwork with literature extracted information

Given the large number of genes and other entities involved in many biological processes, even the most stringently-defined subnetworks can be large and difficult to manually inspect. In our host-virus study, for example, there are more than two thousand hits and interfaces (i.e., genes that are surely involved in HIV replication). Here we present a methodology for generating a *view* of a subnetwork, which is a graphical representation of the part of an inferred subnetwork that is highly related to a given concept of interest. The concept might represent, for example, a process, subcellular location, or stage of the viral lifecycle.

Given a subnetwork and a set of genes *N*^*Q*^ representing a concept of interest, the view generation process returns a set of nodes, and associated edges and paths, that are enriched for gene set. The set of genes could be specified (i) manually, (ii) by selecting genes that have been annotated with an ontology term of interest, or (iii) by identifying genes associated with the concept of interest in the scientific literature. Here we explore the third approach by using queries to GADGET to define gene sets that are closely related to specific concepts.

To compute a view, we first rank every node *n* in the inferred subnetwork for its predicted functional similarity to the given query set of genes *N*^*Q*^. Nodes are considered functionally similar if they share relevant paths. Our similarity function, $s(n,N^{Q})$, measures the fraction of paths in an inferred subnetwork that contain both *n* and at least one query node $q\in N^{Q}$, out of all paths that contain either *n* or any query node *q*. Let $P_{c}\left(n\right)$ be the set of paths in the consensus network that contain a node *n*. Our similarity function is defined as:

$$s\left(n,N^{Q}\right)=\frac{\left|P_{c}\left(n\right)\;\cap \;(\cup_{q\in N^{Q}}P_{c}\left(q\right))\right|}{\left|P_{c}\left(n\right)\;\cup \;(\cup_{q\in N^{Q}}P_{c}\left(q\right))\right|}$$

After ranking the consensus nodes by this similarity function, we take the top *k* as predicted additions to the query set. We then extract all of the paths that consist exclusively of query nodes, predicted additions, and targets.

## Results/discussion

### HIV host-virus subnetwork inference

In the HIV subnetwork application, our baseline set of source nodes were those genes identified as important to HIV replication in RNAi studies. Here consider the effect of augmenting this set of source nodes with human genes returned by GADGET for the query *HIV*. We evaluated the resulting subnetworks by measuring their ability to predict the relevance of genes to HIV replication as determined by whether they were included in an inferred subnetwork.

We used a methodology in which information about the relevance of 25% of the hits and interfaces (i.e., known relevant genes) was held aside on each iteration. That is, although the genes were still included in the background network, information about whether they were hits, interfaces, or neither was hidden. We can estimate the accuracy of our approach by checking each inferred subnetwork for the presence of the hits and interfaces that have been held aside from their input. Given the absence of a set of genes that known not to be involved in HIV replication, we used the set of all unconfirmed background-network human genes as the set of negatives. For each gene, we calculated a confidence value as its frequency of being included in the inferred subnetworks when held aside. By varying a threshold on these confidence values, we plotted a precision-recall curve characterizing the predictive accuracy of our method. Recall is defined as the fraction of truly relevant genes (hits and interfaces) that are predicted to be relevant, and precision is defined as the fraction of genes predicted to be relevant that truly are relevant. In this context, we consider precision to be the more important of the two measures, as it is better to avoid devoting follow-up experiments to false positives.

**Fig 6:**
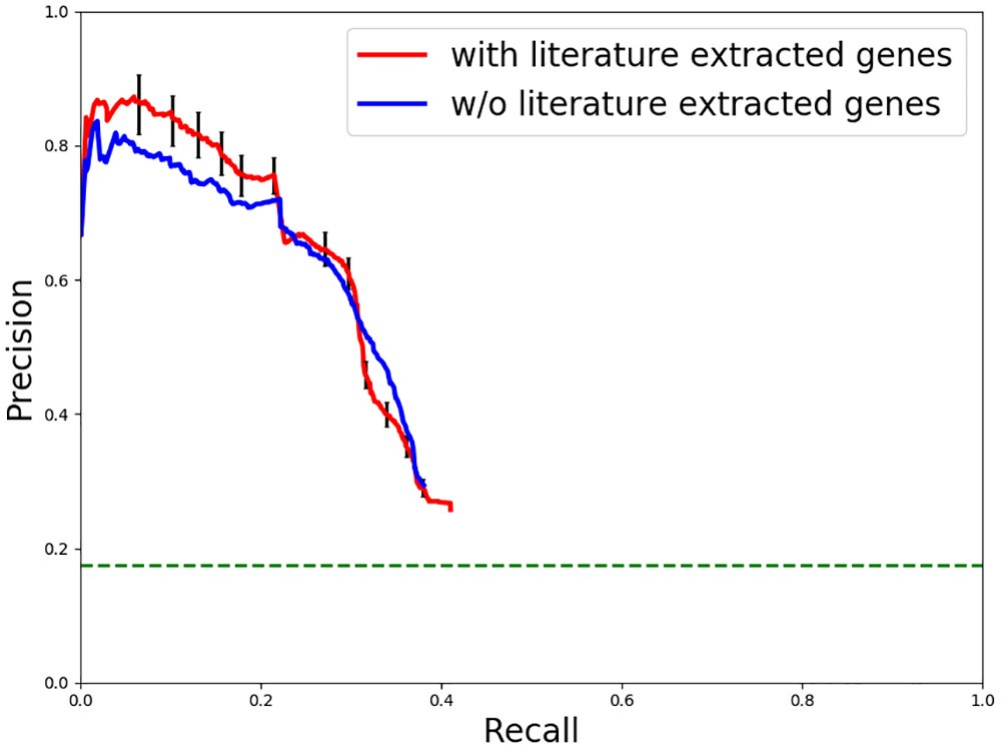
Precision-recall curves for HIV replication subnetworks. The blue line represents results from the consensus network *without* GADGET genes augmenting the sources. The red line represents results from the consensus network generated with GADGET augmentation. The horizontal light green line in the figure represents the prevalence of known hits and interfaces in the background network.

We inferred consensus subnetworks and generated precision-recall curves for both the baseline approach and the GADGET-augmented approach. These results are shown in Fig 6. The horizontal green line in the figure represents the prevalence of known hits and interfaces in the background network, and thus represents the default level of precision. Although both approaches demonstrate substantial predictive accuracy, the GADGET-augmented subnetworks show a significant increase in precision at the high-confidence (low recall) end of the curves, demonstrating the value of incorporating literature-extracted information into the process.

**Fig 7:**
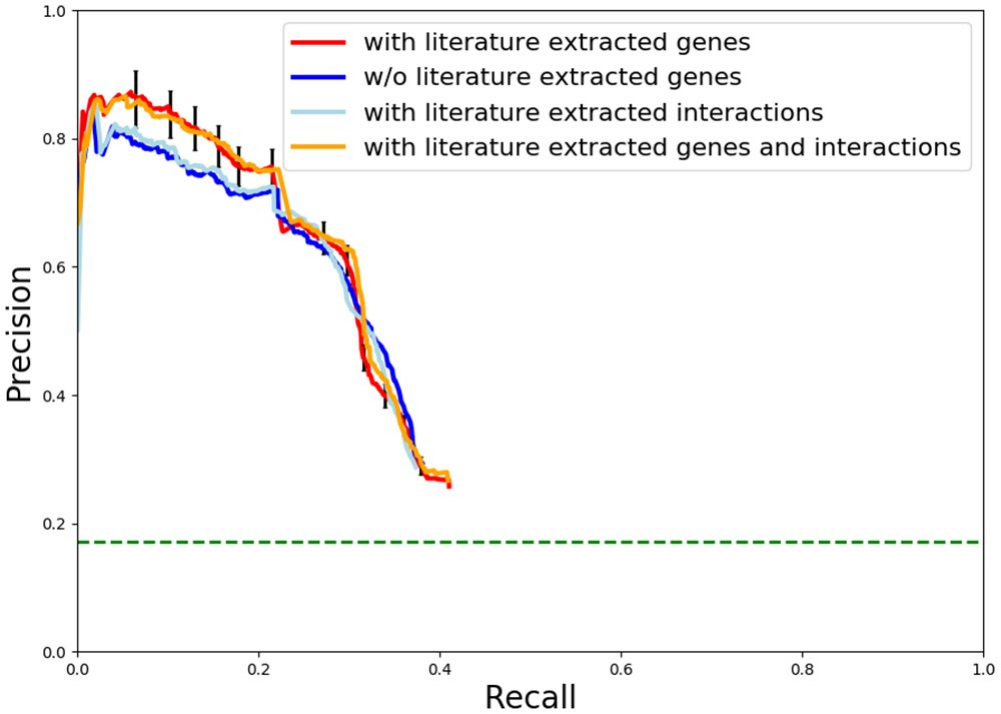
Precision-recall curves for HIV replication subnetworks with and without literature extracted genes and interactions. The blue line represents results from the consensus network without GADGET or Literome augmentation. The red line represents results from the consensus network generated with GADGET genes augmenting the sources. The light blue line represents results from the consensus network generated with Literome interactions augmenting the network. The orange line represents results from the consensus network generated with both GADGET genes and Literome interactions augmenting the sources. The horizontal light green line in the figure represents the prevalence of known hits and interfaces in the background network.

We also considered augmenting our background networks with interactions extracted from the scientific literature. We queried Literome with our list of relevant genes and returned all interactions that contained at least one of those genes. We then incorporated these interactions into our baseline background network. Comparing the precision-recall curves from the baseline approach and the Literome-augmented approach, there was no significant improvement in precision at any point along the curve. We also added literature-extracted interactions to our GADGET-augmented background network. When comparing the precision-recall curves from the GADGET-augmented approach and the GADGET and Literome augmented approach, there was once again no significant improvement in precision. However, we note that the addition of the Literome interactions did not diminish the accuracy of the inferred subnetworks, suggesting that there is little risk of overfitting when including literature extracted interactions. Fig 7 shows the precision recall curves for all the augmentation experiments performed.

### NR2F1 subnetwork inference

For the breast cancer task, our goal was to infer a parsimonius subnetwork that connects the nuclear receptor NR2F1 to a set of genes that are differentially expressed (DE) when NR2F1 is overexpressed. Our IP attempts to find the paths connecting NR2F1 to as many differentially expressed genes as possible. With our baseline background network (which does not include edges from Literome), we were able to reach 314 out of 340 differentially expressed genes. In order to reach more DE genes, we used Literome to incorporate additional interactions into the background network. We queried Literome for our 340 DE genes and found all interactions that included one of those genes as a downstream target. We re-ran our IP and discovered that we were subsequently able to reach 326 out of 340 differentially expressed genes from NR2F1. The number of intermediate nodes used to connect the genes also increased when we used Literome due to the fact that more nodes were necessary to reach the additional differentially expressed genes.

**Fig 8:**
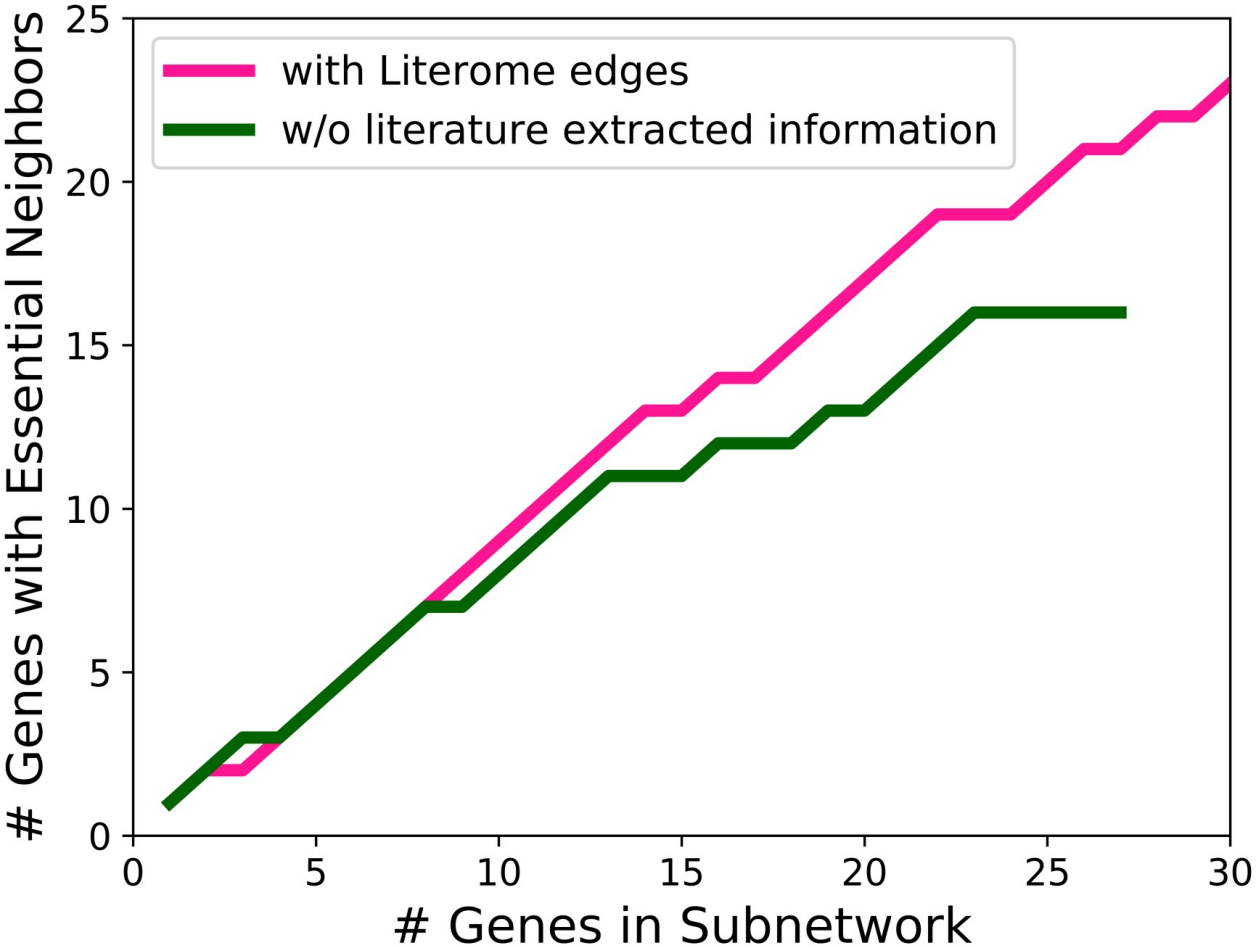
The number of subnetwork genes that are within one interaction away from a set of known essential basal cell genes from Marcotte et al. [45]. The magenta line represents genes included the Literome-augmented subnetworks, and the green line represents genes included in the baseline subnetworks which did not contain literature extracted information.

We use a set of genes that are essential for basal tumor cell generation [45] to determine the degree to which our IP returns a subnetwork that is biologically relevant. We expect that the inferred subnetwork will be closely related to genes involved in basal tummor cell generation. Although none of these genes is incorporated in our consensus subnetworks, a number of them are neighbors (in the background network) with genes in the inferred subnetworks. Fig 8 displays the cumulative number of subnetwork genes that are neighbors of a gene essential for basal tumor cell generation. We rank each gene included in a subnetwork ensemble by the number of subnetworks in which it occurs, and construct these plots for our baseline subnetwork ensemble and our subnetwork ensemble that used Literome interactions. When they incorporate regulatory interactions from Literome, our inferred subnetworks are more related to the basal tumor cell essential genes. The larger number of genes that are incorporated into some member of the Literome-based ensemble is due to the fact that the constituent subnetworks tend to be larger since they connect to more DE genes.

**Fig 9:**
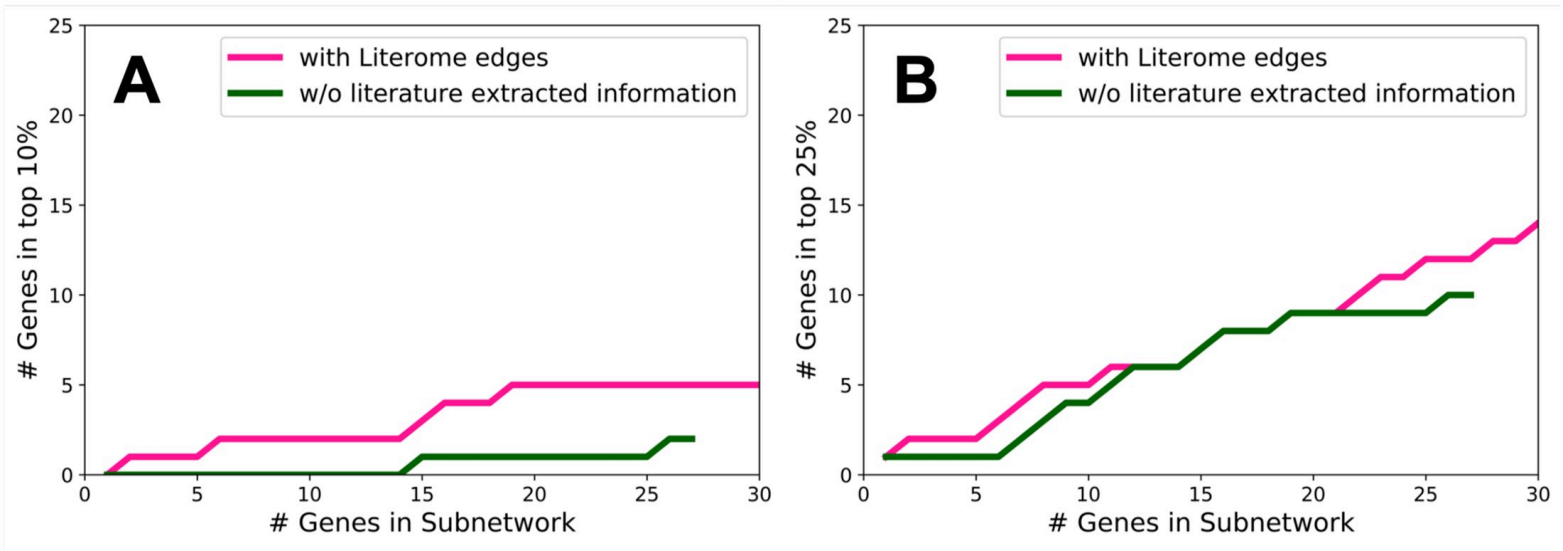
The number of subnetwork genes that are in (A) the top 10% in RNA-Seq abundance, and (B) the top 25% in RNA-Seq abundance. The magenta line represents genes included in the Literome-augmented subnetwork, and the green line represents genes included in the baseline subnetwork which did not contain literature extracted information.

We also use RNA-Seq data to evaluate the subnetworks. Genes that are highly expressed may be more essential for the differences within certain cell types. We obtained RNA-Seq data in counts per million (CPM) for every gene expressed in our cells and ranked the genes based on sequence read abundance. Similar to the analysis above, we varied a threshold on the ranked list of genes and counted the number of genes above the threshold that were in the top 10% of genes in terms of RNA-Seq abundance. In Fig 9, we see that more subnetwork genes are in the top ten percent of the highly expressed genes when we use Literome edges.

The results of this study indicate that literature-extracted interactions may provide value by enabling an inferred subnetwork to “explain” additional data. In this application, we were able to include more DE genes into the inferred subnetwork when including literature-extracted interactions in our background network. Moreover, the augmentation with literature-extracted interactions led to the incorporation of additional relevant genes in the inferred subnetwork.

### Generating views of the HIV host-virus inferred subnetwork

To demonstrate the value of our view-generation approach in browsing and understanding a large subnetwork, we consider the case of computing views onto our inferred HIV consensus subnetwork. This subnetwork includes 14,426 edges connecting 948 nodes and thus is quite large and complex to comprehend. The inherent complexity of HIV subnetwork is due to the fact that a large number of host genes and processes are involved across the multiple stages of viral replication. We illustrate the view-generation approach by computing views that focus on genes related to the concepts of *membrane scission* and *intrinsically disordered proteins*. Whereas the inferred subnetwork represents the process of HIV replication, the views we generate isolate more specific concepts (membrane scission, intrinsically disordered proteins) represented within the subnetwork.

In order to assemble a set of genes for the *membrane scission* view, we issued the query *HIV AND “membrane scission”* to GADGET. This query returns 16 genes which GADGET has determined are associated with the query. We used our similarity function to rank all nodes in the HIV consensus subnetwork based on the frequency with which they are found in the same paths as these GADGET query genes. We took the top 10 of these ranked genes to be used as predicted additions to the membrane scission concept. We then assembled the consensus paths that consisted entirely of our GADGET query genes, predicted additions, and HIV proteins.

**Fig 10:**
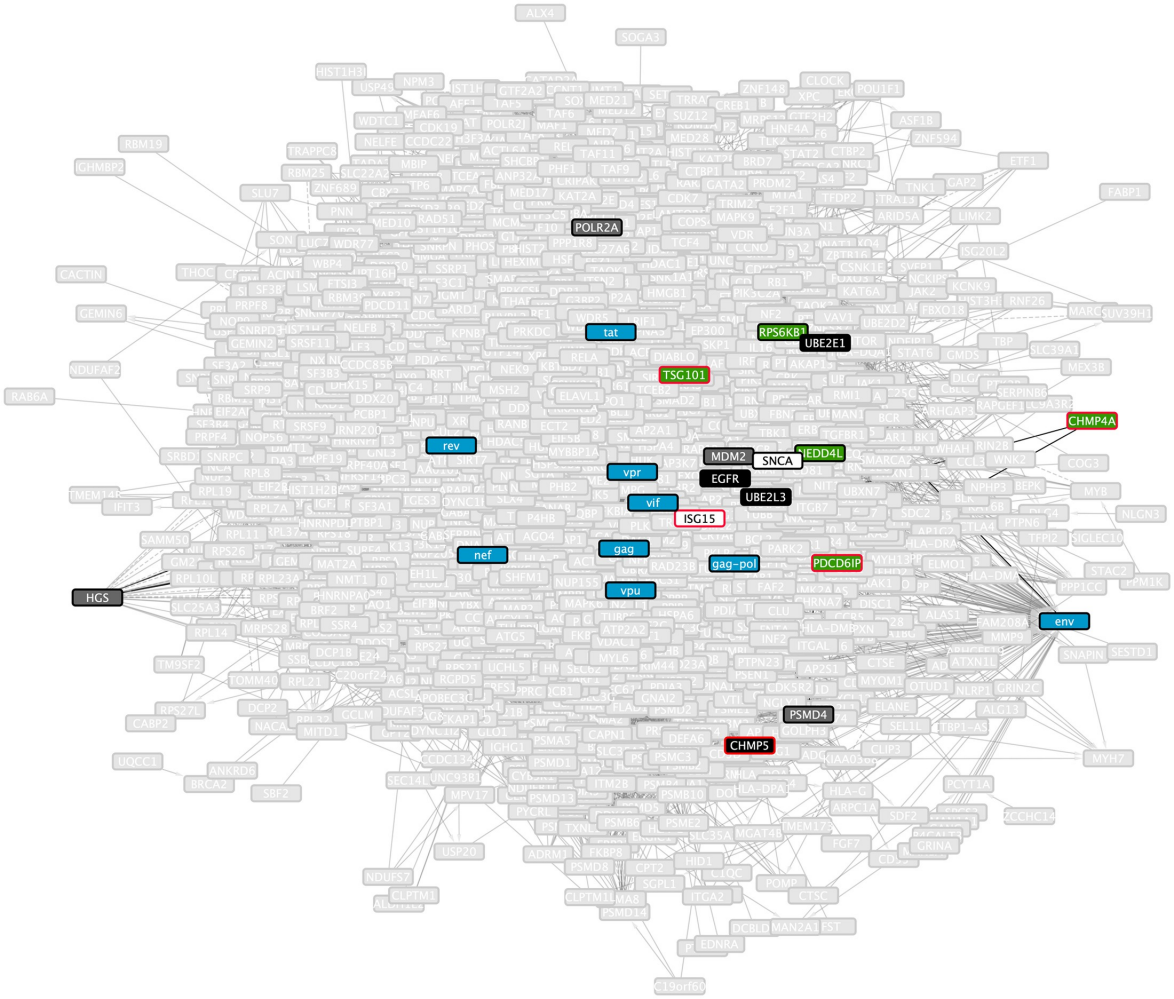
Graphical representation of the inferred HIV subnetwork and the elements of it that are selected for the *membrane scission* view. Light grey nodes represent nodes within the subnetwork that are not part of the view. Black nodes represent RNAi hits (source nodes), green nodes represent host interface genes, dark grey nodes represent elements that are both RNAi hits and interfaces, and blue nodes represent viral components (target nodes). Nodes with red borders are the GADGET hits used to anchor the view.

**Fig 11:**
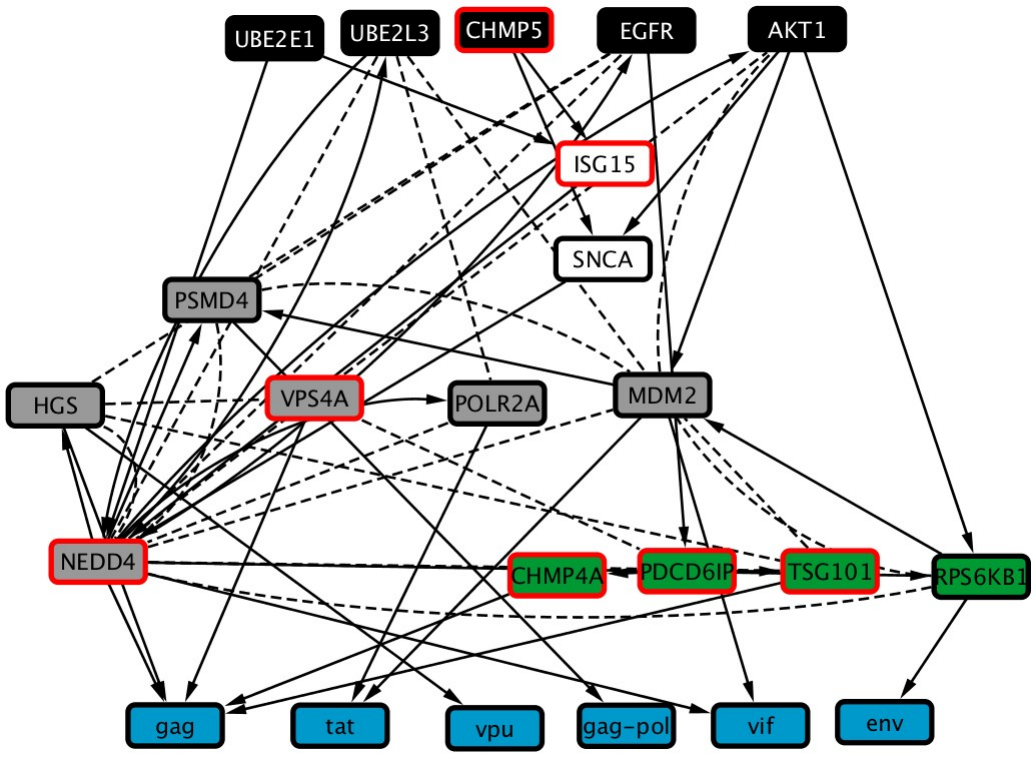
Graphical representation of a view onto the HIV subnetwork for the *membrane scission* concept. Black nodes represent RNAi hits (source nodes), green nodes represent host interface genes, grey nodes represent elements that are both RNAi hits and interfaces, and blue nodes represent viral components (target nodes). Nodes with red borders are the GADGET hits used to anchor the view.

Fig 10 shows the complete inferred HIV subnetwork, highlighting the genes that are selected for the *membrane scission* view. Fig 11 shows the resulting view for this concept. Seven of the 16 genes returned by the GADGET query are present in it. Four of the additional genes included are RNAi hits, one is an interface, and four are both hits and interfaces. As illustrated by this example, views provide a flexible and concise way to comprehend a large subnetwork by selecting conceptually coherent portions of it.

Although the gene set that served as the basis of this view came from a GADGET query, there are multiple sources which could provide a gene set such as the Gene Ontology [46]. The advantage of GADGET in this context is that it can retrieve a list of genes for a very specific concept (note that our query specified the conjunction of *HIV* and *“membrane scission”*), or a concept that is not defined in any ontology.

**Fig 12:**
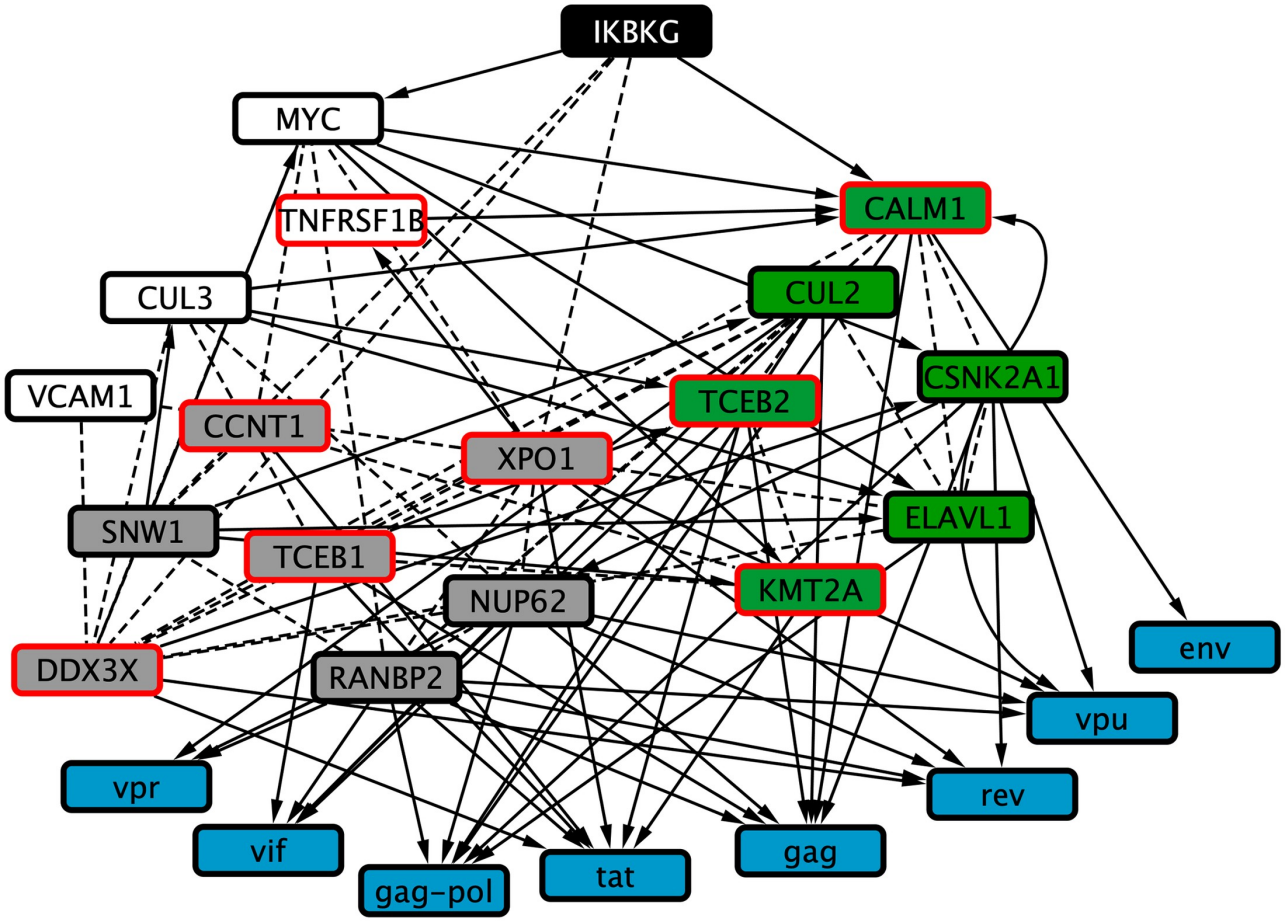
Graphical representation of a view onto the HIV subnetwork for the concept *intrinsically disordered proteins*. Black nodes represent RNAi hits (source nodes), green nodes represent host interface genes, grey nodes represent elements that are both RNAi hits and interfaces, and blue nodes represent viral components (target nodes). Nodes with red borders are the GADGET hits used to anchor the view.

In order to demonstrate that queries are not limited to terms defined in an ontology, we computed a view based on the concept *intrinsically disordered proteins*. We queried GADGET for the terms *HIV AND “intrinsically disordered.”* Using the same methodology as above, we inferred a view that includes of 8 of the 36 genes returned by GADGET. The view, which is shown in Fig 12 consists of 26 nodes connected by 94 edges.

The experiments presented in this section show how literature-extracted information can be used to explore and gain insight into a large inferred subnetwork by generating different views of the subnetwork. The key idea of a view is to select a subset of the genes in an inferred subnetwork that are enriched for a concept of interest, such as a cellular process or gene products sharing a specific physical property.

### Conclusion

We have investigated the use of information automatically extracted from the scientific literature to augment the process of inferring subnetworks characterizing biological responses of interest. Specifically, we have used literature-extracted information to (i) enlarge the set of nodes identified as sources in a subnetwork inference task, (ii) enlarge the set of interactions used in the background network, and (iii) support targeted browsing of a large inferred subnetwork by computing *views* of the subnetwork that are closely related to concepts of interest.

The empirical studies we present demonstrate that literature-extracted information can improve the explanatory power and accuracy of subnetwork inference in both of the applications considered. However, we argue that our general approaches are relevant to a range of other network analysis tasks, including predicting and ranking genes that are likely to be involved in the same response as a given set of genes [47, 48, 49, 50, 51, 52], and combining multiple network data sets in order to perform classification or collaborative recommendation [53, 54, 55, 56, 57].

Although the specific constraints and objective functions used in subnetwork inference are somewhat application-dependent, the integer programs typically incorporate several common elements. Among these elements are a background network consisting of subcellular entities and interactions, and a procedure that aims to connect source and target nodes in the network in order to optimize various objectives. Our approach is applicable to any network analysis task that shares these common elements. In cases in which either the source or target nodes are believed to be incomplete, tools like GADGET can be used to augment the sources or targets by mining the scientific literature. In cases in which the relevant interactions are believed to be incomplete, tools like Literome can be used to augment the set of interactions in the background network.

We consider this work as an initial foray into exploring the range of ways in which text mining can boost the subnetwork-inference process. For example, we also plan to explore using literature-extracted information to extend the types of relationships that are represented in the background network, and to prioritize the inclusion of entities and interactions into subnetworks.

## References

[1] Stark C, Breitkreutz BJ, Reguly T, Boucher L, Breitkreutz A, Tyers M. BioGRID: a general repository for interaction datasets. Nucleic Acids Research. 2006;34(Database issue):D535–D539. 10.1093/nar/gkj109 16381927 PMC1347471

[2] Croft D, Mundo AF, Haw R, Milacic M, Weiser J, Wu G, et al The Reactome pathway knowledgebase. Nucleic Acids Research. 2014;42(Database issue):D472–D477. 10.1093/nar/gkt1102 24243840 PMC3965010

[3] Chasman D, Gancarz B, Hao L, Ferris M, Ahlquist P, Craven M. Inferring host gene subnetworks involved in viral replication. PLoS Computational Biology. 2014;10(5):e1003626 10.1371/journal.pcbi.1003626 24874113 PMC4038467

[4] Chasman D. Improving the interpretability of integer linear programming methods for biological subnetwork inference. Department of Computer Sciences, University of Wisconsin Madison, WI; 2014.

[5] Smits BM, Haag JD, Rissman AI, Sharma D, Tran A, Schoenborn AA, et al The gene desert mammary carcinoma susceptibility locus Mcs1a regulates Nr2f1 modifying mammary epithelial cell differentiation and proliferation. PLoS Genetics 2013;9(6):e1003549 10.1371/journal.pgen.1003549 23785296 PMC3681674

[6] Liang S, Fuhrman S, Somogyi R. Reveal, a general reverse engineering algorithm for inference of genetic network architectures. Pacific Symposium on Biocomputing. 1998;3:18–29. 9697168

[7] Akutsu T, Miyano S, Kuhara S. Identification of genetic networks from a small number of gene expression patterns under the Boolean network model. Pacific Symposium on Biocomputing. 1999; p. 17–28. 10380182 10.1142/9789814447300_0003

[8] Ideker TE, Thorsson V, Karp RM. Discovery of regulatory interactions through perturbation: inference and experimental design. Pacific Symposium on Biocomputing. 2000;5:305–316. 10.1142/9789814447331_0029 10902179

[9] Reiser PGK, King RD, Kell DB, Muggleton SH, Bryant CH, Oliver SG. Developing a logical model of yeast metabolism. Linköping Electronic Articles in Computer and Information Science. 2001;6(24).

[10] Yeang CH, Ideker T, Jaakkola T. Physical network models. Journal of Computational Biology. 2004;11(2-3):243–262. 10.1089/1066527041410382 15285891

[11] Markowetz F, Bloch J, Spang R. Non-transcriptional pathway features reconstructed from secondary effects of RNA interference. Bioinformatics. 2005;21(21):4026–4032. 10.1093/bioinformatics/bti662 16159925

[12] Tamaddoni-Nezhad A, Chaleil R, Kakas A, Muggleton S. Application of abductive ILP to learning metabolic network inhibition from temporal data. Machine Learning. 2006;64(1-3):209–230. 10.1007/s10994-006-8988-x

[13] Ourfali O, Shlomi T, Ideker T, Ruppin E, Sharan R. SPINE: a framework for signaling-regulatory pathway inference from cause-effect experiments. Bioinformatics. 2007;23(13):i359–i366. 10.1093/bioinformatics/btm170 17646318

[14] Medvedovsky A, Bafna V, Zwick U, Sharan R. An algorithm for orienting graphs based on cause-effect pairs and its applications to orienting protein networks. In: Proceedings of the 8th International Workshop on Algorithms in Bioinformatics. Springer-Verlag; 2008. p. 222–232.

[15] Vaske CJ, House C, Luu T, Frank B, Yeang CH, Lee NH, et al A factor graph nested effects model to identify networks from genetic perturbations. PLoS Computational Biology. 2009;5(1):e1000274 10.1371/journal.pcbi.1000274 19180177 PMC2613752

[16] Peleg T, Yosef N, Ruppin E, Sharan R. Network-free inference of knockout effects in yeast. PLoS Computational Biology. 2010;6(1):e1000635 10.1371/journal.pcbi.1000635 20066032 PMC2795781

[17] Maeyer DD, Renkens J, Cloots L, Raedt LD, Marchal K. PheNetic: network-based interpretation of unstructured gene lists in E. coli. Molecular BioSystems. 2013;9(7):1594–1603. 10.1039/c3mb25551d 23591551

[18] Hao L, He Q, Wang Z, Craven M, Newton MA, Ahlquist P. Limited agreement of indepdendent RNAi screens for virus-required host genes owes more to false-negative than false-positive factors. PLoS Computational Biology. 2013;9(9):e1003235 10.1371/journal.pcbi.1003235 24068911 PMC3777922

[19] Cherry S, Doukas T, Armknecht S, Whelan S, Wang H, Sarnow P, et al Genome-wide RNAi screen reveals a specific sensitivity of IRES-containing RNA viruses to host translation inhibition. Genes & Development. 2005;19(4):445–452. 10.1101/gad.1267905 15713840 PMC548945

[20] Kushner DB, Lindenbach BD, Grdzelishvili VZ, Noueiry AO, Paul SM, Ahlquist P. Systematic, genome-wide identification of host genes affecting replication of a positive-strand RNA virus. Proceedings of the National Academy of Sciences USA. 2003;100(26):15764–15769. 10.1073/pnas.2536857100 PMC307642 14671320

[21] Zhang R, Miner J, Gorman M, Rausch K, Ramage H, White J, et al A CRISPR screen defines a signal peptide processing pathway required by flaviviruses. Nature. 2016;535:164–168. 10.1038/nature18625 27383988 PMC4945490

[22] Brass A, Dykxhoorn D, Benita Y, Yan N, Engelman A, Xavier R, et al Identification of host proteins required for HIV infection through a functional genomic screen. Science. 2008;319:921–926. 10.1126/science.1152725 18187620

[23] Konig R, et al Global analysis of host-pathogen interactions that regulate early-stage HIV-1 replication. Cell. 2008;135(1):49–60. 10.1016/j.cell.2008.07.032 18854154 PMC2628946

[24] Zhou H, Xu M, Huang Q, Gates AT, Zhang XD, Castle JC, et al Genome-scale RNAi screen for host factors required for HIV replication. Cell Host & Microbe. 2008;4(5):495–504. 10.1016/j.chom.2008.10.004 18976975

[25] Yeung ML, Houzet L, Yedavalli VSRK, Jeang KT. A genome-wide short hairpin RNA screening of Jurkat T-cells for human proteins contributing to productive HIV-1 replication. Journal of Biological Chemistry. 2009;284(29):19463–19473. 10.1074/jbc.M109.010033 19460752 PMC2740572

[26] Liu L, Oliveira NMM, Cheney KM, Pade C, Dreja H, Bergin AMH, et al A whole genome screen for HIV restriction factors. Retrovirology. 2011;8:94 10.1186/1742-4690-8-94 22082156 PMC3228845

[27] Fu W, Sanders-Beer BE, Katz KS, Maglott DR, Pruitt KD, Ptak RG. Human immunodeficiency virus type 1, human protein interaction database at NCBI. Nucleic Acids Research. 2009;37(Database issue):D417–D422. 10.1093/nar/gkn708 18927109 PMC2686594

[28] Newman RH, Hu J, Rho HS, Xie Z, Woodard C, Neiswinger J, et al Construction of human activity-based phosphorylation networks. Molecular Systems Biology. 2013;9:655 10.1038/msb.2013.12 23549483 PMC3658267

[29] Schaefer MH, Fontaine JF, Vinayagam A, Porras P, Wanker EE, Andrade-Navarro MA. HIPPIE: Integrating protein interaction networks with experiment based quality scores. PLoS ONE. 2012;7(2):e31826 10.1371/journal.pone.0031826 22348130 PMC3279424

[30] Prasad TSK, Goel R, Kandasamy K, Keerthikumar S, Kumar S, Mathivanan S, et al Human protein reference database–2009 update. Nucleic Acids Research. 2009;37(Database issue):D767–D772. 10.1093/nar/gkn892 18988627 PMC2686490

[31] Hofmann T, Schölkopf B, Smola AJ. Kernel methods in machine learning. The Annals of Statistics. 2008; p. 1171–1220. 10.1214/009053607000000677

[32] Danna E, Fenelon M, Gu Z, Wunderling R. Generating multiple solutions for mixed integer programming problems. In: Proceedings of the 12th International Conference on Integer Programming and Combinatorial Optimization. Springer-Verlag; 2007. p. 280–294.

[33] Ziegler M, Kiblawi S, Lucas M, Stewart R, Craven M. GADGET: A tool for identifying associations between biomedical concepts, genes and metabolites. Submitted.

[34] Aesoy R, Clyne CD, Chand AL. Insights into orphan nuclear receptors as prognostic markers and novel therapeutic targets for breast cancer. Frontiers in Endocrinology. 2015;6:115 10.3389/fendo.2015.00115 26300846 PMC4528200

[35] Kittler R, Zhou J, Hua S, Ma L, Liu Y, Pendleton E, et al A comprehensive nuclear receptor network for breast cancer cells. Cell Reports. 2013;3(2):538–551. 10.1016/j.celrep.2013.01.004 23375374

[36] ENCODE Project Consortium and others. An integrated encyclopedia of DNA elements in the human genome. Nature. 2012;489(7414):57–74. 10.1038/nature11247 22955616 PMC3439153

[37] Robinson M, McCarthy D, Smyth G. edgeR: A Bioconductor package for differential expression analysis of digital gene expression data. Bioinformatics. 2010;26(1):139–140. 10.1093/bioinformatics/btp616 19910308 PMC2796818

[38] Ananiadou S, Pyysalo S, Tsujii J, Kell D. Event extraction for systems biology by text mining the literature. Trends in Biotechnology. 2010;28(7):381–390. 10.1016/j.tibtech.2010.04.005 20570001

[39] Li C, Liakata M, Rebholz-Schuhmann D. Biological network extraction from scientific literature: state of the art and challenges. Briefings in Bioinformatics. 2014;15(5):856–877. 10.1093/bib/bbt006 23434632

[40] Pyysalo S, Ohta T, Rak R, Rowley A, Chun HW, Jung SJ, et al Overview of the cancer genetics and pathway curation tasks of BioNLP shared task 2013. BMC Bioinformatics. 2015;16:S2 10.1186/1471-2105-16-S10-S2 26202570 PMC4511510

[41] Chen H, Sharp BM. Content-rich biological network constructed by mining PubMed Abstracts. BMC Bioinformatics. 2004;5:147 10.1186/1471-2105-5-147 15473905 PMC528731

[42] Percha B, Altman R. A global network of biomedical relationships derived from text. Bioinformatics. 2018;34(15):2614–2624. 10.1093/bioinformatics/bty114 29490008 PMC6061699

[43] Poon H, Quirk C, DeZiel C, Heckerman D. Literome: PubMed-scale genomic knowledge base in the cloud. Bioinformatics. 2014;30(19):2840–2842. 10.1093/bioinformatics/btu383 24939151

[44] Poon H, Toutanova K, Quirk C. Distant supervision for cancer pathway extraction from text. In: Pacific Symposium on Biocomputing; 2015 p. 120–131. 25592574

[45] Marcotte R, Sayad A, Brown KR, Sanchez-Garcia F, Reimand J, Haider M, et al Functional genomic landscape of human breast cancer drivers, vulnerabilities, and resistance. Cell. 2016;164(1):293–309. 10.1016/j.cell.2015.11.062 26771497 PMC4724865

[46] The Gene Ontology Consortium. The Gene Ontology in 2010: extensions and refinements. Nucleic Acids Research. 2010;38:D331–D335. 10.1093/nar/gkp1018 19920128 PMC2808930

[47] Köhler S, Bauer S, Horn D, Robinson PN. Walking the interactome for prioritization of candidate disease genes. American Journal of Human Genetics. 2008;82(4):949–958. 10.1016/j.ajhg.2008.02.013 18371930 PMC2427257

[48] Navlakha S, Kingsford C. The power of protein interaction networks for associating genes with diseases. Bioinformatics. 2010;26(8):1057–1063. 10.1093/bioinformatics/btq076 20185403 PMC2853684

[49] Vanunu O, Magger O, Ruppin E, Shlomi T, Sharan R. Associating genes and protein complexes with disease via network propagation. PLoS Computational Biology. 2010;6(1):e1000641 10.1371/journal.pcbi.1000641 20090828 PMC2797085

[50] Chen Y, Jiang T, Jiang R. Uncover disease genes by maximizing information flow in the phenome-interactome network. Bioinformatics. 2011;27(13):i167–i176. 10.1093/bioinformatics/btr213 21685067 PMC3117332

[51] Murali TM, Dyer MD, Badger D, Tyler BM, Katze MG. Network-based prediction and analysis of HIV dependency factors. PLoS Computational Biology. 2011;7(9):e1002164 10.1371/journal.pcbi.1002164 21966263 PMC3178628

[52] Börnigen D, Tranchevent LC, Bonachela-Capdevila F, Devriendt K, Moor BD, Causmaecker PD, et al An unbiased evaluation of gene prioritization tools. Bioinformatics. 2012;28(23):3081–3088. 10.1093/bioinformatics/bts581 23047555

[53] Pavlidis P, Weston J, Cai J, Noble WS. Learning gene functional classifications from multiple data types. Journal of Computational Biology. 2002;9(2):401–411. 10.1089/10665270252935539 12015889

[54] Kato T, Tsuda K, Asai K. Selective integration of multiple biological data for supervised network inference. Bioinformatics. 2005;21(10):2488–2495. 10.1093/bioinformatics/bti339 15728114

[55] Tsuda K, Shin H, Schölkopf B. Fast protein classification with multiple networks. Bioinformatics. 2005;21(Suppl 2):ii59–ii65. 10.1093/bioinformatics/bti1110 16204126

[56] Lippert C, Ghahramani Z, Borgwardt KM. Gene function prediction from synthetic lethality networks via ranking on demand. Bioinformatics. 2010;26(7):912–918. 10.1093/bioinformatics/btq053 20154010

[57] Zitnik M, Zupan B. Matrix factorization-based data fusion for gene function prediction in baker’s yeast and slime mold. Pacific Symposium on Biocomputing. 2014; p. 400–411. 24297565 PMC3902649

